# Depression with and without Comorbid Substance Dependence in a Child Welfare Sample of Young Adults

**DOI:** 10.1155/2011/475248

**Published:** 2010-12-09

**Authors:** Heather Orton Anderson, Anne M. Libby

**Affiliations:** ^1^School of Pharmacy, University of Colorado, Nighthorse Campbell Native Health Building, Mail Stop F800, P.O. Box 6508, Aurora, CO 80045, USA; ^2^School of Pharmacy, University of Colorado, 12631 E. 17th Avenue, Campus Box C238-L15, Aurora, CO 80045, USA

## Abstract

The objective of this study was to estimate the prevalence of depression with and without substance dependence and examine the effect of risk factors on subsequent disorders among a cohort of young adults in the US Child Welfare System (CWS). We used longitudinal data for 834 young adults age 18–21 from the National Survey of Child and Adolescent Well-being. Depressive symptoms and substance use were measured at baseline (age 11–15); diagnoses of depression and substance dependence were identified at the last wave of data collection (age 18–21). Likelihood of subsequent depression with or without substance dependence was three times higher for those with clinically significant depressive symptoms at baseline. Frequent use of substances at baseline significantly increased the likelihood of subsequent depression with comorbid substance dependence compared to depression alone. These results support screening youth in the CWS at younger ages for both depressive symptoms and substance use with the hope that these disorders can be detected earlier.

## 1. Introduction

The US Child Welfare System (CWS) sees a large number of youth with mental health problems because they have often experienced maltreatment and other risk factors such as poverty and separation from caregivers [1–3]. A study of youth age 2 to 14 years (*n* = 3,803) in the US CWS estimated that nearly half had significant emotional or behavioral problems upon entering the system [3]. A smaller study of 426 youth age 6 to 18 years in the CWS in San Diego County estimated the prevalence of major depression to be 4.7% [4]. 

Depression and substance use disorders are known to occur together more often than not [5–8]. In clinical samples, comorbid depression is seen in 70–80% of adolescents with substance use disorder [9]. In studies of depression, substance use is often found to significantly increase the risk of depression. One such study found that among youth in grades 6, 8, and 10 (*n* = 9,863), substance use significantly increased the risk of depression, with relative risks ranging from 2.5 to 3.1 depending on the type of substance used [10]. Comorbid depression and substance use disorder is a risk factor for poor treatment outcomes, treatment drop-out, and earlier relapse [11]. Youth with depression and comorbid substance use disorder also have an increased risk for suicidal behaviors, including ideation, attempt, and completed suicide [12]. 

The estimate of major depression prevalence among youth in the San Diego County CWS (4.7%) resulted from a multisector study in San Diego County which randomly selected youth age 6 to 18 years who were active in at least one of the following systems: Child Welfare, Substance Abuse programs, Juvenile Justice, Specialty Mental Health, and Public-School programs for youth with serious emotional disturbance. While the prevalence of major depression among youth in Child Welfare was 4.7%, it was estimated to be nearly double (8.3%) among youth in Substance Abuse programs [4]. Focusing on youth aged 13 to 18 in the San Diego County multisector study, lifetime and past year nontobacco substance use disorder were estimated to be 19.2% and 11.0%, respectively, among youth in Child Welfare [13]. The prevalence of lifetime and past year substance use disorder among youth in the Specialty Mental Health sector was more than double (40.8% and 22.9%, respectively). Although comorbidity of depression and substance use disorder was not directly reported, these results support a possible co-occurrence in these youth [4, 13]. 

A recent study used data from the National Survey of Child and Adolescent Well-Being (NSCAW) to describe depressive symptoms and substance use among youth in the CWS [14]. Among a sample of 1,179 NSCAW youth aged 11 to 15 years at baseline, 9% had clinically significant depressive symptoms, 5% had problems with frequent substance use, and 4% had comorbid depressive symptoms and frequent substance use. Depressive symptoms were measured using the Children's Depression Inventory (CDI), and frequent substance use was based on the youth self-report of type and frequency of substances used in the past 30 days. The study found that clinically significant depressive symptoms were more than twice as common in girls as boys (17% versus 7%, *P* < .05), but there were no gender differences in the prevalence of past month substance use. Differences in depression symptoms and substance use severity were found between groups; boys with clinically significant depressive symptoms and frequent substance use had more severe depression symptoms compared to boys with clinically significant depressive symptoms without frequent substance use.

Two main developmental hypotheses attempt to explain the comorbidity between depression and substance use disorders. First, depression develops as a consequence of a substance use disorder; and second, depression precedes the substance use disorder [8]. The latter is often referred to as the self-medication hypothesis and has found less support in the current literature. For example, a study using AddHealth examined the ordering of depression symptoms and tobacco use and found a significant association where tobacco use preceded subsequent depressive symptoms; the reverse association was not supported [15]. A longitudinal study of Australian school-aged youth found that marijuana use strongly predicted later depression; again, the reverse relationship was not supported [16]. 

Depression and substance use disorder in youth are both more likely in the presence of stressors and share many risk factors [8]. Having a caregiver with a mental health problem has been found to increase the risk of the youth having emotional or substance use problems themselves [17–20]. A prior study using NSCAW found a significant association between caregiver depression and substance use problems and youth internalizing problems [2]. Child maltreatment in general is known to increase the likelihood of both depression and substance use disorder in youth [21–23]. Type of maltreatment has also been found to be associated with these mental health problems. One study in particular found that sexual abuse and neglect significantly increased the risk of substance use disorder among a sample of youth in the CWS in San Diego County [24]. Other risk factors unique to youth in the CWS are placement instability and number of out-of-home placements, which have been found to be associated with well-being outcomes [25], behavior problems [26], child maladjustment [27], and substance use disorder [24]. 

NSCAW has paved the way for filling gaps in child welfare mental health research by providing a large, nationally representative sample of youth in the US CWS with a wide range of outcomes and risk factors measured longitudinally for youth and their families. However, until the most recent (fifth) wave of data collection, no diagnostic instrument for obtaining clinical indications of depression or substance use disorder was included in the youth interview. Therefore, all estimates of depression and substance use disorders have relied on instruments such as the Child Behavior Checklist, the CDI, and self-report of substance use. Wave 5 of NSCAW data collection included a young adult interview given to the cohort of NSCAW youth who were age 18 or older by April 20, 2006. This interview included an instrument that resulted in a clinical diagnosis of both depression and substance dependence not previously available for this cohort. 

The goal of the current study was to build on prior work of the authors [14] and use the newest data from Wave 5 of NSCAW to fill the following gaps: (1) estimate the prevalence of major depression with and without comorbid substance dependence among young adults in NSCAW, (2) estimate the effect of depressive symptoms and frequent substance use at baseline of NSCAW on subsequent depression with and without substance dependence, and (3) examine the effect of other possible risk factors measured at baseline of NSCAW on subsequent depression with and without comorbid substance dependence. Risk factors were chosen based on those found to be associated with depression and/or substance use disorder in the current literature, and particularly those specific to children in the child welfare system: caregiver mental health and substance use problems, out-of-home placements, and type of maltreatment experienced.

The authors hypothesized that depressive symptoms and patterns of substance use at baseline would be significantly associated with the presence of a subsequent clinical diagnosis of depression and/or substance dependence. Specifically, the authors sought to determine whether baseline depressive symptoms and substance use would increase the likelihood of subsequent comorbid diagnoses among youth with subsequent depression. Results from this study will help focus screening and intervention efforts of children as they enter and navigate the CWS.

## 2. Materials and Methods

### 2.1. Study Cohort

Data for this study were drawn from NSCAW, a longitudinal study of youth and their families who came into contact with the US CWS between October 1999 and December 2000. At baseline (Wave 1), 5,501 youth aged birth to 15 years were interviewed, as were their caseworker, primary caregiver, and teacher (if applicable). The youth were then followed for up to four additional waves of data collection: 12-month followup (Wave 2), 18-month followup (Wave 3), 36-month followup (Wave 4), and a final wave of data collection based on age cohort (Wave 5). Wave 5 included the Young Adult Interview, a special instrument administered to youth who had turned 18 years old by April 2006. Data for the current study were drawn from the youth, caregiver and caseworker interviews at baseline, and the Young Adult Interview at Wave 5.

The current study was built on a previous study for which the focus was the comorbidity of depression symptoms and substance use at baseline among a cohort of 1,179 NSCAW youth age 11 to 15 years [14]. The Young Adult Interview administered at Wave 5 did not include the same measurements of depressive symptoms and substance use as available at baseline but did include measurements of clinical depression and alcohol and drug dependence. From the previous study's cohort of 1,179, a total of 834 youth had aged into the young adult cohort at Wave 5 and were administered the diagnostic instruments; the current study included these 834 youth. The number of months between the baseline interview and the Wave 5 Young Adult Interview ranged from 64 to 93 (mean = 74 months). Compared to the 345 youth excluded, the study cohort (*n* = 834) had significantly more females and significantly higher depressive symptoms at baseline. The groups did not differ with respect to any other baseline characteristics.

### 2.2. Measures

*Depressive symptoms* were measured at baseline for youth age 7 and older through administration of the Children's Depression Inventory (CDI) [28]. According to a meta-analysis of 310 datasets which included 61,424 youth age 8 to 16 (29,637 boys), the CDI is the most commonly used instrument to assess depression in youth [29]. The CDI includes 27 items addressing a range of depressive symptoms, each answered on a 3-point scale (0 = absence of symptom, 1 = mild symptom, 2 = definite symptom). The sum of all 27 responses was used to create a total raw score, which was converted to a standardized *t*-score ranging from 0 to 100 using the standardized tables in Kovacs' CDI Profile Form [28]. Internal consistency for the CDI was examined for the NSCAW sample and found to be good, with a mean internal consistency of 0.81 for youth age 7 to 12 years old and 0.87 for 13 to 15 years old [30]. A total score of 66 and greater was used to indicate *clinically significant depressive symptoms*, as done by the experts involved in the development of NSCAW [30].

*Substance use (type and frequency)* was measured at baseline for youth age 11 years and older through a set of questions regarding lifetime and past month use of alcohol, tobacco, and other substances. In order to assure confidentiality, these items were administered using an Audio Computer-Assisted Self-Interview program, which allowed the youth to answer the substance use items without the interviewer knowing their answers. Youth were first asked if they had used a particular substance in their lifetime. Those who indicated they had used that substance at least once were then asked on how many days they had used that substance in the past 30 days (none, 1 day, 2 days, 3–5 days, 6–11 days, 12–19 days, and 20 or more days). The youth were asked about each of the following substances: tobacco (cigarettes and chewing tobacco); alcohol; marijuana; inhalants (glue, gasoline, and other liquids and gases); hard drugs (cocaine, crack, and heroin); nonmedical use of prescription medications such as pain killers, tranquilizers, stimulants, and sedatives. 

Since a clinical interview that would result in a diagnosis of substance dependence was not administered to the youth at baseline, severity of substance use was instead characterized using the number of days in the past month substances were used. Specifically, *frequent substance use (FSU)* was defined as use of one or more substances, including tobacco, for 20 or more days in the past month.

*Depression symptom and FSU comorbidity* was identified at baseline using four mutually exclusive groups: neither clinically significant depressive symptoms (CDI score ≥ 66) nor FSU; clinically significant depressive symptoms (CDI score ≥ 66) only; FSU only; clinically significant depressive symptoms (CDI score ≥ 66) comorbid with FSU.

*Diagnoses of major depression and substance dependence *in the past 12 months were obtained for young adults at Wave 5 through administration of a structured clinical interview using the Composite International Diagnostic Interview—Short Form (CIDI-SF) [31]. The CIDI-SF resulted in diagnoses for major depression, alcohol dependence, and drug dependence, each based on DSM-IV criteria. Diagnoses of alcohol and drug abuse were not assessed with the CIDI-SF. Alcohol dependence and drug dependence were assessed separately; a participant was considered to have substance dependence if either alcohol dependence or drug dependence was present. Drugs included in the dependence diagnosis were inhalants, marijuana, cocaine, heroin, hallucinogens, and prescription medications without a prescription, such as sedatives, tranquilizers, amphetamines/stimulants, and prescription pain killers. Tobacco was not included in the substance dependence diagnosis.

*Caregiver mental health and substance use problems* were assessed at baseline through both caregiver self-report and caseworker report. Caregivers were administered the CIDI-SF [31] to assess major depression and substance dependence. In addition to these clinical diagnoses, caseworkers indicated whether or not the caregiver had serious emotional problems or substance abuse problems at baseline. These two sources of data (caregiver report and caseworker report) were combined to create a summary indication of caregiver depression or mental health problems and a summary indication of caregiver substance use problems at baseline. For each, if either the caregiver self-report of the caseworker report indicated the problem, the combined summary variable was set to present.

*Demographic characteristics of the youth* included gender, age (in years at baseline), and race/ethnicity (White, Black, Hispanic, and other (including American Indian and Asian)). Placement of the youth (in-home versus out-of home) was known at baseline; a count of out-of-home placements for the youth between baseline and Wave 5 was also available. The type(s) of maltreatment experienced by the child and included in the caseworker's initial report to the CWS was indicated by the caseworker at baseline using a modified Maltreatment Classification Scale [32]. Categories included physical abuse, sexual abuse, emotional abuse, physical neglect (i.e., failure to provide), nonphysical neglect (i.e., failure to supervise), and other types of neglect (i.e., abandonment, moral or legal maltreatment, educational maltreatment, and exploitation). More than one type of maltreatment could have been reported for each youth.

### 2.3. Statistical Analysis

The NSCAW study design included a two-stage, complex sampling process in which the US was first divided into strata, and then each stratum was divided into primary sampling units. In order to adjust for this complex sample design and calculate population-based estimates representative of the youth in the US CWS, analysis weights and other study design variables were incorporated into all analyses, which were completed using STATA 10 [33]. Additional detailed information about the NSCAW study design and weight derivation is published elsewhere [34, 35].

Characteristics of the study cohort were described and compared by depression and substance dependence status at Wave 5 using weighted chi-square tests and t-tests. Separate weighted multivariate logistic regression models were used to estimate the conditional likelihood of subsequent depression (with or without substance dependence) compared to no depression at Wave 5, and the conditional likelihood of depression without substance dependence compared to depression with substance dependence at Wave 5. The final multivariate models reported included demographic variables regardless of significance, and other variables found to be statistically significant at *P* < .05. All percentages, odds ratios, confidence intervals, and statistical tests reported are weighted, while all sample sizes are reported as unweighted.

## 3. Results and Discussion

### 3.1. Description of Study Cohort

The study cohort included 834 youth age 11 to 15 years old (mean = 12.8 years) at baseline of NSCAW; ages ranged from 18 to 21 years old at Wave 5 (mean = 19.1 years). Slightly more than half the cohort (58%) was male. The cohort was ethnically diverse, with 50% being White, 28% being Black, 15% being Hispanic, and 7% being of another race/ethnic group. The majority of the cohort was living at home at baseline (87%); 82% had no out-of-home placements between baseline and their young adult survey, which occurred between 64 and 93 months following baseline (mean = 74.5 months). At baseline, the caseworkers reported all types of maltreatment that had been reported in the initial investigation; 41% of the youth had been physically abused, 12% had been sexually abused, 14% had been emotionally abused, 19% had been physically neglected, 38% had experienced nonphysical neglect, and the remaining 16% were reported to have experienced some other form of maltreatment or neglect. The mean number of different types of maltreatment reported per youth was 1.4, indicating some had experienced more than one type of maltreatment prior to the investigation.

### 3.2. Depression and Substance Dependence

**Figure 1:**
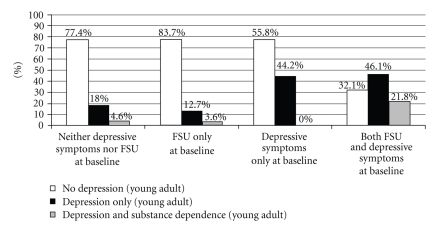
Prevalence of depression with or without substance dependence as a young adult by depressive symptoms and substance use at baseline.

Twenty-six percent (*n* = 208) of the study cohort had major depression at Wave 5 (21% without substance dependence; 5% comorbid with substance dependence). At baseline, depressive symptoms and substance use were assessed through self-report. Based on prior work [14], youth in the current study cohort were identified as being in one of four groups at baseline: clinically significant depressive symptoms only (10.0% at baseline), frequent substance use (FSU) only (5.3% at baseline), both clinically significant depressive symptoms and FSU (4.3% at baseline), or neither clinically significant depressive symptoms nor FSU (80.4% at baseline). For each of these four groups, prevalence of major depression with and without substance dependence at Wave 5 is presented in Figure 1. Prevalence of depression without substance dependence as a young adult was highest in the youth who had clinically significant depressive symptoms with or without FSU at baseline. Prevalence of depression with substance dependence as a young adult was highest in the group with both clinically significant depressive symptoms and FSU at baseline. The overall association between depressive symptoms and FSU at baseline and subsequent depression with or without substance dependence was statistically significant (*χ* ^2^(6) = 490.53, *P* < .01).

### 3.3. Unadjusted Associations

Unadjusted associations between baseline characteristics of the youth and their caregivers and subsequent depression with and without substance dependence are presented in Table 1. There were significantly more girls with subsequent depression (with or without substance dependence) compared to no subsequent depression (*P* < .05). There was no significant effect of gender, however, on having subsequent depression without substance dependence compared to subsequent depression comorbid with substance dependence. FSU (20 + days in the past 30 days) at baseline was seen significantly more in the group with subsequent depression (with or without substance dependence) for all substances except tobacco and marijuana. Frequent substance use at baseline also significantly differentiated between youth with subsequent depression without substance dependence compared to youth with both depression and substance dependence. Youth with comorbid depression and substance dependence were significantly more likely to have used alcohol, inhalants, hard drugs, and prescription medication for nonmedical purposes at baseline than those with depression only. Baseline depressive symptoms were also significantly associated with subsequent depression. Among the youth with subsequent depression (with or without substance dependence), 28% had clinically significant depressive symptoms at baseline, compared to only 10% of youth without subsequent depression (*P* < .01). Baseline depressive symptoms were not significantly associated with subsequent depression alone versus subsequent depression comorbid with substance dependence.

Type of maltreatment experienced by the youth at baseline was not significantly associated with subsequent depression with or without substance dependence. Being in an out-of home placement at baseline or any time between baseline and Wave 5 was also not associated with depression (with or without substance dependence) or associated with depression without substance dependence versus depression comorbid with substance dependence (see Table 1). However, the number of out-of-home placements between baseline and Wave 5 was significantly associated with depression without substance dependence versus depression comorbid with substance dependence. Youth with subsequent comorbid depression and substance dependence had significantly *fewer* out-of-home placements between baseline and Wave 5 (mean = 1.8) compared to those with subsequent depression without substance dependence (mean = 3.2; *P* < .05). 

Having a caregiver with a substance use problem at baseline was also significantly associated with comorbid depression and substance dependence at Wave 5. Significantly fewer youth with subsequent depression and substance dependence had a caregiver at baseline with a substance use problem compared to youth with subsequent depression without substance dependence. These unadjusted results were further explored using multivariate logistic regression (see Table 2).

### 3.4. Adjusted Associations

The likelihood of subsequent depression (with or without substance dependence) and of subsequent depression comorbid with substance dependence, conditional on baseline depressive symptoms and substance use, was estimated using multivariate logistic regression (see Table 2). Age, gender, and race/ethnicity of the youth were adjusted for in the multivariate models. After adjusting for these baseline characteristics, number of out-of-home placements and caregiver substance use problems at baseline no longer had a significant effect on subsequent depression comorbid with substance dependence and were therefore excluded from the final multivariate model reported in Table 2. However, likelihood of depression with or without substance dependence (compared to no depression) was nearly three times higher for those with clinically significant depressive symptoms at baseline than those without clinically significant depressive symptoms (OR = 2.6, *P* < .01); baseline depressive symptoms did not have a significant effect on a subsequent depression diagnosis comorbid with substance dependence compared to a depression diagnosis only. Frequent use of alcohol at baseline also significantly increased the likelihood of subsequent depression with or without substance dependence (OR = 21.0, *P* < .01). Frequent use of any nontobacco substance at baseline significantly increased the likelihood of subsequent depression with comorbid substance dependence compared to depression alone (OR = 37.2, *P* < .01).

## 4. Conclusions

Prevalence of major depression among the current cohort of young adults age 18 to 21 years at Wave 5 of NSCAW was 26%; 18% of these youth also had comorbid substance dependence. In the National Comorbidity Study (NCS), past year major depression was seen in 21.2%, 11.7%, and 15.6% of young adults and adults age 17 to 18, 19 to 20, and 21 to 22 years, respectively [36]. Prevalence of major depression in the past 12 months was estimated to be 6.6% for adults age 18 years and older in the NCS-R; 8.5% of those with 12-month major depressive disorder also had comorbid substance dependence in the past 12 months [37]. Prevalence of major depression overall, and prevalence of comorbid substance dependence among those with major depression, was higher in the current study's cohort of young adults from NSCAW. This is consistent with other studies in the literature that have found more depression and emotional problems among youth in the CWS. The only other study known to have estimated the prevalence of major depression among youth in the CWS included youth age 6 to 18 years old in the San Diego County CWS and found a much lower prevalence of depression, only 4.7% [4]. However, this number is difficult to compare to the current study because of the age difference between the two study cohorts. 

Results of this study support the idea that some aspects of the CWS may in fact be protective against disorders among youth in the system [38]. Youth with a caregiver with substance use problems at baseline were significantly less likely to have depression with substance dependence, compared to depression only, at Wave 5 of NSCAW. The number of out-of-home placements was also found to be protective, with more out-of-home placements being significantly associated with a decreased risk of depression comorbid with substance dependence compared to depression alone. It's important to note that these significant associations were unadjusted; after other covariates such as age, gender, and baseline depressive symptoms and substance use were included in the model, these associations were no longer significant. However, they are important to consider when planning future research and interventions. Youth with caregivers who have a substance use problem when they enter the system may be more visible and therefore have a higher chance of receiving preventive services throughout their experience in the system. 

A similar argument may be made for number of out-of-home placements. The more often the youth moves in the system, the more visible they are to the caseworkers and the more likely they may be to get preventive or treatment services. The CWS is thought to be a gateway to service for these reasons [38]. However, the association between out-of-home placements and substance use disorders among youth has been somewhat inconsistent in research to date. One study of youth age 13 to 18 years in the San Diego County CWS found a marginal association between number of out-of-home placements and severity of substance use problems [24]. Another study of adolescents in foster care found no association between placements and drug use [39]. These inconsistent results support the need for further research on the impact of out-of-home placements on subsequent disorders in youth.

Multivariate models found that youth with clinically significant depressive symptoms at baseline were significantly more likely to have major depression at Wave 5, but such depressive symptoms did not make those youth more or less likely to have depression and substance dependence compared to depression alone. These results were adjusted for baseline substance use and demographic correlates such as gender, age and race/ethnicity. Frequent alcohol use significantly increased the risk of subsequent depression, while frequent use of any nontobacco substance significantly increased the risk of depression comorbid with substance dependence versus depression alone among those youth with major depression at Wave 5. 

This study found that youth who presented to the CWS with clinically significant depressive symptoms were more likely to have a subsequent diagnosis of depression. Other studies have made similar conclusions. One such study found that adolescent depression significantly predicted adult depression, but that the effect was accounted for by other comorbid diagnoses such as substance use disorder [40]. In the current study, youth who presented with frequent use of nontobacco substances were more likely to have subsequent comorbid depression and substance dependence. With both depressive symptoms and substance use at baseline significantly predicting subsequent clinical disorder, the time after the youth enter the CWS presents a window of opportunity for intervention and treatment that may decrease the likelhood of these youth having subsequent disorders.

### 4.1. Strengths and Limitations

The limitations of this study are tied to the challenges associated with using pre-existing data such as that from NSCAW. The instruments administered to the youth at baseline did not include a diagnostic assessment of depression or substance dependence. Substance use was measured through self-report as type and frequency during lifetime and the past 30 days. Depressive symptoms were measured using a validated instrument, the CDI, which provided a guideline for identifying youth with clinically significant depressive symptoms but did not provide an actual diagnosis based on DSM criteria. The addition of the CIDI-SF to the young adult interview at Wave 5 provided a subsequent clinical diagnosis of major depression and substance dependence. However, because substance *abuse* was not included in the diagnosis, this study may have underestimated the scope of substance use disorders in this population. 

The young adults who received the CIDI-SF at Wave 5 did not also receive the interview section on type and frequency of substances use, or the CDI, so direct correlations between the two types of measurements could not be investigated. We were, however, able to estimate the association between the baseline measurements of depressive symptoms and substance use with Wave 5 diagnoses of major depression and substance dependence, which is not known to have been done before.

Compared to the 345 subjects excluded, the study cohort (*n* = 834) had significantly more females and significantly higher depressive symptoms at baseline. The groups did not differ with respect to any other baseline characteristics. These results indicate that the 834 young adults included in the current study may have been more likely to have depression at Wave 5 because they had higher depressive symptoms scores at baseline and included more females. However, it is unknown how the exclusion of the 345 subjects may have biased the results. Another limitation results from the small sample size among the youth with depression at Wave 5; only 35 youth had comorbid depression and substance dependence, possibly resulting in a lack of power to detect significant associations among that group.

Other limitations of the current study arise from measures not included in analyses. Reports of services received by the youth are collected from the caseworker, caregiver, and youth at each wave of NSCWA data collection. However, these services cannot be tied to specific diagnoses. Therefore, treatment received by the youth was not controlled for in the current analyses. Although an in-depth analysis of the services received by youth with depression and/or substance dependence is possible, it is outside the scope of the current study. Also excluded from this study were measures of external behaviors, such as conduct disorder, which are known to be associated with substance use disorders among young adults [41, 42]. Although outside the scope of the current study, further research that considers externalizing disorders as a possible moderator or mediator of the association between baseline depressive symptoms and substance use and subsequent clinical diagnoses is warranted.

NSCAW is a large, nationally representative sample of youth in the US CWS and provides numerous measurements for youth and their caregivers. The availability of child maltreatment data reported by the caseworker at baseline avoided having to rely on self-report from the youth or their caregiver. The cohort included in this study was old enough at baseline to report their own substance use and depressive symptoms, so the less reliable caregiver reports did not need to be used. Overall, NSCAW provides a unique, large dataset with numerous opportunities to explore mental health among youth in the child welfare system.

### 4.2. Policy Implications

A recent systematic review for the US Preventive Services Task Force concluded that regular screening of adolescents for depression could lead to increased and earlier diagnosis and greater improvement of symptoms [43]. The results of the current study support that conclusion, finding a significant association between depressive symptoms at baseline of NSCAW and subsequent major depression. Further, youth who reported frequent use of nontobacco substances at baseline were significantly more likely to have both depression and substance dependence at Wave 5 compared to depression alone. Although these results cannot be generalized to youth outside the US Child Welfare System, they lend support to the idea that screening youth at younger ages for both depression symptoms and substance use may lead to prevention or earlier detection of depression and substance disorders. Substance use disorders and depression are often comorbid, and treatment of one may lead to improvement of the other [44, 45]. By screening for both substance use and depression when youth are admitted to the Child Welfare System, the problems with which the youth are struggling at that time can be identified and appropriate help can be made available.

## Figures and Tables

**Table 1:** Associations between baseline youth and caregiver characteristics and subsequent depression (*n* = 834).

	Total sample		Depression	
	(*n* = 834)		(*n* = 208)	
				Depression
	No		Depression	+ substance
	depression	Depression ^a^	only	dependence
	(*n* = 626)	(*n* = 208)	(*n* = 173)	(*n* = 35)
*Youth demographic characteristics *				
Age in years at baseline (range 11–15)				
Mean (SE)	12.80 (0.08)	12.92 (0.14)	12.85 (0.16)	13.21 (0.32)
Female (%)	54.33	69.75*	71.56	61.72
Race/ethnicity (%)				
White	49.23	53.27	49.56	68.96
Black	30.83	18.17	19.81	11.24*
Hispanic	13.91	17.13	17.77	14.44
Other	6.04	11.43	12.86	5.37
Initial type of maltreatment (%)				
Physical abuse	43.6	35.2	32.43	45.6
Sexual abuse	11.54	14.42	12.06	23.3
Emotional abuse	14.38	14.08	15.14	10.09
Physical neglect	17.85	23.05	25.75	12.93
Neglect	36.46	41.17	43.54	32.25
Out-of-home placement				
At baseline (%)	12.07	13.94	11.75	23.69
Ever (baseline to W5) (%)	16.25	22.16	20.14	30.37
If ever had OOH placement:				
Mean no. placements (SE)	2.75 (0.16)	2.80 (0.41)	3.18 (0.50)	1.78 (0.34)*
Range	1–11	1–18	1–18	1–7
*Caregiver characteristics at baseline *				
Substance use problem (%)	15.52	9.48	10.96	2.77*
Depression/MH problem (%)	37.02	38.4	41.37	25.13
*Youth substance use and depressive symptoms*				
Substance use at baseline (% who reported 20+ days in past 30 days)				
Alcohol	0.14	3.86**	0.12	20.55**
Tobacco	8.06	14.08	12.12	22.56
Marijuana	1.09	4.52	0.67	21.06**
Inhalants	0.19	3.61**	0.001	18.87**
Hard drugs	0.13	3.57**	0	18.87**
Nonmedical use of Rx	0.11	3.58**	0	18.87**
Mean no. substances used (SE)	0.09 (0.02)	0.32 (0.20)	0.12 (0.05)	1.20 (0.91)
FSU at baseline (%)	7.91	14.34	12.36	23.13
Clinically significant depressive sx at baseline (%)	9.46	27.62**	29.48	19.29

Abbreviations: SUD: substance use disorder; Rx: prescription; FSU: frequent substance use; sx: symptoms.

**P < .01; *P < .05.

^a^ Depression with and without substance dependence.

**Table 2:** Conditional likelihood of subsequent depression with or without substance dependence.

	Depression with or without substance dependence^a^		Depression with comorbid substance dependence^b^	
	OR	(95% CI)	OR	(95% CI)
Model *N*	793		205	
*Youth demographic characteristics *				
Age in years at baseline	1.01	(0.81, 1.25)	1.1	(0.65, 1.86)
Female	1.64	(0.88, 3.06)	1.22	(0.29, 5.16)
Race/ethnicity				
White (referent group)				
Black	0.7	(0.29, 1.70)	0.53	(0.11, 2.63)
Hispanic	1.26	(0.47, 3.38)	0.75	(0.11, 4.96)
Other	2.02	(0.84, 4.82)	0.19	(0.02, 1.93)
*Youth substance use and depressive sympyoms *				
Substance use at baseline (20+ days in past 30)				
Alcohol	21.03	(2.22, 199.22)**	—	
Any nontobacco substance	—		37.25	(3.33, 416.54)**
Clinically significant depressive sx at baseline	2.63	(1.08, 6.38)**	—	

Abbreviation: sx: symptoms.

— Not included in model.

**OR significantly different from 1.0, *P* < .01.

^a^Referent group: no depression.

^b^Referent group: deprssion without substance dependence.
